# Color Blindness

**Published:** 1857-04

**Authors:** 


### EDITORIAL DEPARTMENT.

*Color Blindness.*—This is a name given by Dr. Wilson, Regius Professor of Technology in the University of Edinburgh, to a remarkable defect of vision of which our readers must have met with numerous instances. Before proceeding to give Dr. Wilson's results, as stated in his recent publication, we may mention a case or two coming within our own knowledge.

Some years since, in one of the rural districts of our state, there resided a gentleman who had a very dim perception of the distinction of colors. All the light hues, he was in the habit of calling *red;* to all the darker tints he gave the name, *blue.* In his own neighborhood, his peculiarity was so well known, that people were able, in some measure, to understand his very remarkable expressions. But on one occasion, a valuable dark bay horse of his, happened to stray away. He pursued him for some distance, and excited no little amusement and wonder in the country town, by inquiring if any one had seen a *blue* horse wandering in that vicinity.

Another instance occurs to us of a little boy, who came running to his mother in a state of great excitement, desiring her to come out to the garden to see a "beautiful red rose." The mother, not aware of having any roses in bloom at the time, went with her son, and was surprised to find his delight occasioned by a full blown yellow marigold.

The eminent chemist, Dalton, was the first who called public attention to this peculiarity, by describing it as it occurred in his own person. Not much attention was paid to it until Dr. Wilson took the matter in hand and made it a subject of serious study. He advertised in the London Athenæum for facts, and in this way obtained from the sufferers themselves accurate accounts of this curious malady. He has classified the cases under three general heads:

1. Inability to distinguish between the minor shades of composite colors, such as browns, grays and neutral tints.

2. Inability to distinguish between the primary colors, red, blue and yellow, or between the secondary and tertiary colors, such as green, purple, orange and brown.

3. Inability to discern any color, properly so called, so that black and white (i. e. light and shade) are the only variations of tint perceived.

The first form is very common, the second cannot be called rare, while the third is but seldom met with. Dr. Wilson has not observed a case. That which comes nearest to it, is that of the physician to whom all colors were alike by day, but who could detect their differences by artificial light.

It is remarkable how long persons may remain in ignorance of this defect. Dr. Wilson gives an example of a journeyman tailor, who gained such a reputation as a cutter and fitter that he was promoted to the station of foreman in the shop. His color-blindness was then soon detected since he was obliged to match cloth for the journeymen. On one occasion he provided a scarlet livery with green strings, and on another informed a customer that a red and blue striped cloth was all blue.

The examination of this defect cannot be considered a mere scientific announcement. It has some most important practical results. A steersman, for example, may imperil the lives of the crew and passengers by reason of this imperfection. For example, the British admiralty directs that all British steamers must show "between sunset and sunrise, a *white* light at the foremast head, a *green* light on the port side and a *red* light on the port side." Now if a steamer lie across the bows of another vessel, it is evident that the color of the lights turned towards him, constitutes the sole guide to the steersman whether he is to go to starboard or to larboard. Should he mistake the color, he must run the steamer down, or should she be in motion, may run the risk of being struck by her bows, when he thinks he is going astern of her.

The same risk exists in regard to railway signals at night. These are usually colored lights, one tint signifying safety and another danger. The color-blind engineer who should mistake the hue of the signal light, might dash his train to pieces and sacrifice many lives. It is therefore essential that these companies should see to the state of the perceptions of the men in their employ, or else should adopt some signal of form or motion, to avoid the errors attendant upon color. The Baltimore and Ohio Railroad company station men along the heavy grades of the Cheat river region. These men are furnished with lights. Should every thing be in proper order, they keep the lanterns stationary. Should there be danger, they move them up and down. The arrangement is as simple as that of the colors and no risk is incurred.

As for the cause of this disease, Dr. Wilson has discovered that it is usually congenital. When acquired, it often depends upon derangement of the liver or stomach and passes off on the disappearance of the primary disease. It has been known to depend upon concussion of the brain combined with long continued cerebral excitement.

